# Cancer Immunotherapy: Harnessing the Immune System to Fight Cancer

**DOI:** 10.3390/jcm11216356

**Published:** 2022-10-27

**Authors:** Alessandro Rizzo, Veronica Mollica, Matteo Santoni, Francesco Massari

**Affiliations:** 1 Struttura Semplice Dipartimentale di Oncologia Medica per la Presa in Carico Globale del Paziente Oncologico “Don Tonino Bello”, I.R.C.C.S. Istituto Tumori “Giovanni Paolo II”, Viale Orazio Flacco 65, 70124 Bari, Italy; 2 Medical Oncology, IRCCS Azienda Ospedaliero-Universitaria di Bologna, Via Albertoni-15, 40138 Bologna, Italy; 3 Department of Experimental, Diagnostic and Specialty Medicine, University of Bologna, 40138 Bologna, Italy; 4 Medical Oncology Unit, Macerata General Hospital, 62100 Macerata, Italy

The advent of cancer immunotherapy has represented an unprecedented revolution in patients with hematological and solid tumors [1,2,3]. Immunotherapy with immune checkpoint inhibitors (ICIs) has been suggested to induce durable and robust anticancer responses in cancer patients [4,5,6]. For example, the treatment landscape for metastatic renal cell carcinoma (mRCC) has changed dramatically over the last decade, with the standard of care shifting from tyrosine kinase inhibitor (TKI) monotherapy to combination treatments, including ICIs [7,8]. In this genitourinary tumor, there are several approved immune-based combinations, including two ICIs or an ICI plus a multitarget TKI, and these treatments have significant clinical benefit compared to monotherapy [9,10,11,12]. Several successful clinical trials have expanded the use of ICIs in an impressive number of solid tumors, ranging from urothelial carcinoma to gastric carcinoma, colorectal cancer, hepatocellular carcinoma, and head and neck squamous cell carcinoma [13,14,15,16,17,18,19]. In addition, following the approval in tissue-agnostic malignancies with microsatellite instability, the PD-1 inhibitor pembrolizumab became the first agent to be selectively approved according to a molecular biomarker rather than by the primary tumor site [20,21].

However, several questions regarding modern immunotherapy remain unanswered. Among these, ICIs present a specific set of treatment-related toxicities, which are commonly known as immune-related adverse events (irAEs) and are a result of the erroneous activation of the immune system against self-antigens [22,23,24]. Several organ systems may be affected by irAEs, including the thyroid, pancreas, and liver, with the incidence and severity of irAEs depending on multiple factors, including the type of ICI, the tumor type, and the disease setting [25,26,27]. Another fundamental issue in current and future cancer immunotherapy is the identification of reliable biomarkers of response or resistance [28,29]. In fact, while ICIs have found their role in several tumors in monotherapy or as part of combinatorial strategies, the lack of validated biomarkers of response represents an important issue, since not all cancer patients benefit from immunotherapy [30,31]. Based on these concepts, a greater understanding of the role of potential biomarkers, including programmed death ligand 1 (PD-L1) expression, tumor mutational burden (TMB), microsatellite instability (MSI) status, gut microbiota, concomitant medications, and several others, is fundamental [32,33,34,35,36,37,38,39,40]. Additionally, clinical trials on cancer immunotherapy frequently differ in terms of drugs, patients, designs, terms of study phases, and inconsistent clinical outcomes.

This Special Issue aims to highlight unanswered questions and future perspectives in modern cancer immunotherapy, including novel immune checkpoint inhibitors and immune-based combinations, mechanisms of action, potential biomarkers predictive of response, experimental treatments, and other timely and emerging topics. International experts in this field will examine key approaches under active investigation in clinical and preclinical research, presenting critical analyses and integrating their own perspectives on promising drugs in clinical trials and the latest therapeutic strategies.
